# Treatment of Multiple Junctional Vertebra Fractures in a Single Case

**DOI:** 10.7759/cureus.13255

**Published:** 2021-02-10

**Authors:** Idris Avci, Salim Senturk

**Affiliations:** 1 Neurosurgery, Mehmet Akif Inan Training and Research Hospital, Sanliurfa, TUR; 2 Neurosurgery, Memorial Bahçelievler Hospital, Spine Center, Istanbul, TUR

**Keywords:** vertebra fracture, posterior stabilization, anterior stabilization, percutaneous stabilization, suicide, junctional fracture

## Abstract

We present a unique case of multiple junctional vertebra fractures in a single patient requiring surgical intervention, the variety of which has not yet been reported in the literature.

A 15-year old female was admitted to our emergency department after a suicide attempt from jumping from the window of a five-floor building. On admission her general status was critical, Glasgow Coma Scale (GCS) was 6, and on painful stimuli she was able to move all four extremities. On her spinal CT, a C1 right arcus fracture, a C7 corpus fracture, an L3 and L5 burst fracture and a right sacrum fracture were detected. The patient also suffered from pneumothorax, pleural effusion and pulmonary contusions. After she was stable and extubated, she did not show any motor or sensory deficits. As the patient still had some pleural effusion and pulmonary contusions, posterior approaches were avoided at first and a C6-T1 anterior stabilization with mini plate-screws was performed. After her pulmonary problems resolved, a series of spinal instrumentation surgeries were performed over the following weeks.

A case like this in which multiple traumatic junctional fractures were treated with different surgical techniques has not been reported in the literature before. It is important to emphasize if and when surgical intervention is needed. A multidisciplinary assessment of trauma surgeons, neurosurgeons and anesthesiologists is vital for forming a further treatment plan.

## Introduction

Determining the exact number of vertebral fractures is difficult because many variables need to be taken into consideration, such as the patient's age, comorbidities, activity level or demographics, which are important factors affecting the incidence rate. It is estimated that 11,000 spinal cord injuries occur every year in the United States [1] and 13-163.4 per million worldwide in developed countries [2]. In this presentation we mainly focus on traumatic fractures, which are mostly due high-energy falls and motor vehicle accidents, which account for nearly equal percentages. van den Berg et al. published a review of 13 studies and reported a bimodal age contribution in traumatic vertebral fractures. In the adolescent population the most common cause was traffic accidents. In the age group of 65 and higher falls were the predominant cause [3]. Most traumatic vertebral fractures occur in the thoracic and lumbar areas [4]. Despite cervical fractures being less common, their consequences are likely to be more severe [5]. We present a unique case of traumatic multiple vertebral fractures in a single patient for which we performed multiple spinal surgery techniques not yet reported in the literature in this kind of variety.

## Case presentation

**Figure 1:**
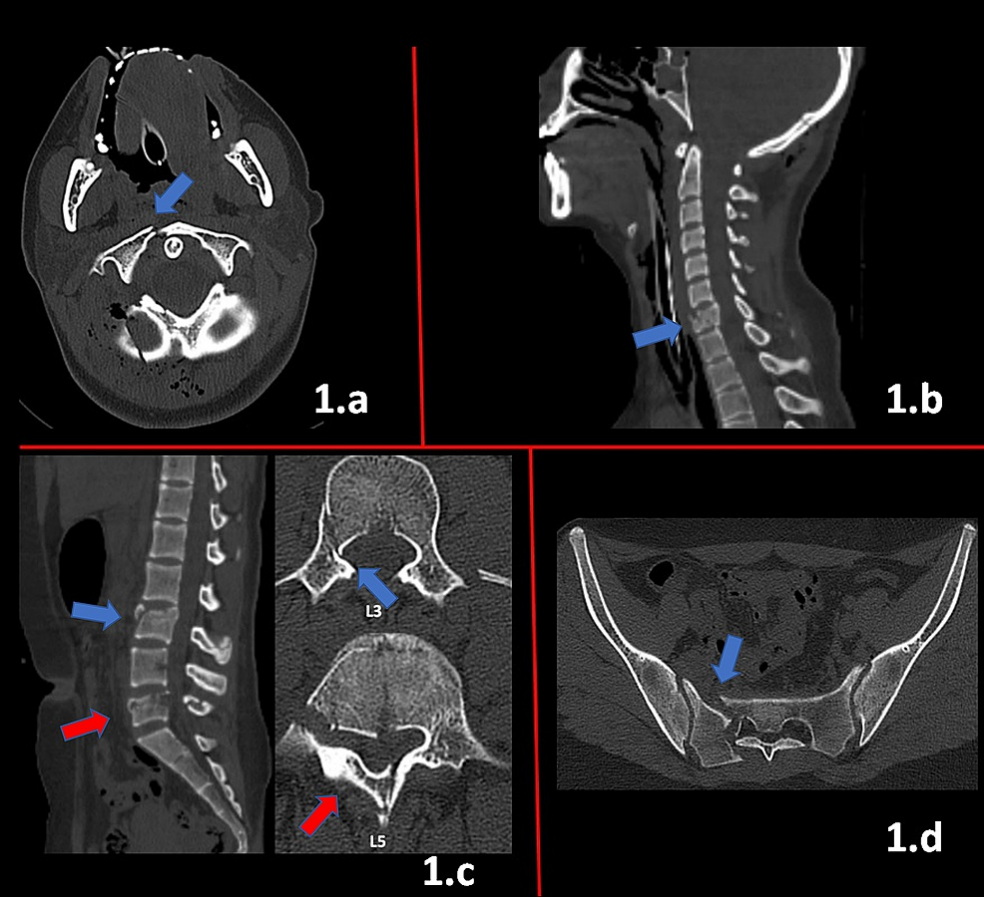
a) cervical CT axial view: right C1 anterior arch fracture (blue arrow), b) cervical CT sagittal view: C7 burst fracture (blue arrow), c) lumbar CT sagittal and axial views of L3 (blue arrow) and L5 burst fractures (red arrow), d) sacrum CT axial view: Denis zone 2 sacral fracture (blue arrow)

A 15-year-old female was admitted to our emergency department after a suicide attempt in which she jumped from the window of a five-floor building. On admission her general status was critical, her Glasgow Coma Scale (GCS) was 6, and on painful stimuli she was able to move all her extremities. She was intubated and transferred to the intensive care unit (ICU). After she was hemodynamically stable, cranial and spinal computed tomography (CT) scans were performed. She had an occipital linear fracture with subarachnoid hemorrhage and cerebral edema on her cranial CT. On her spinal CT a right C1 arcus fracture (Figure 1A), a C7 corpus fracture (Figure 1B), L3 and L5 burst fractures (Figure 1C) and a right sacral fracture (Figure 1D) were detected. 

**Figure 2:**
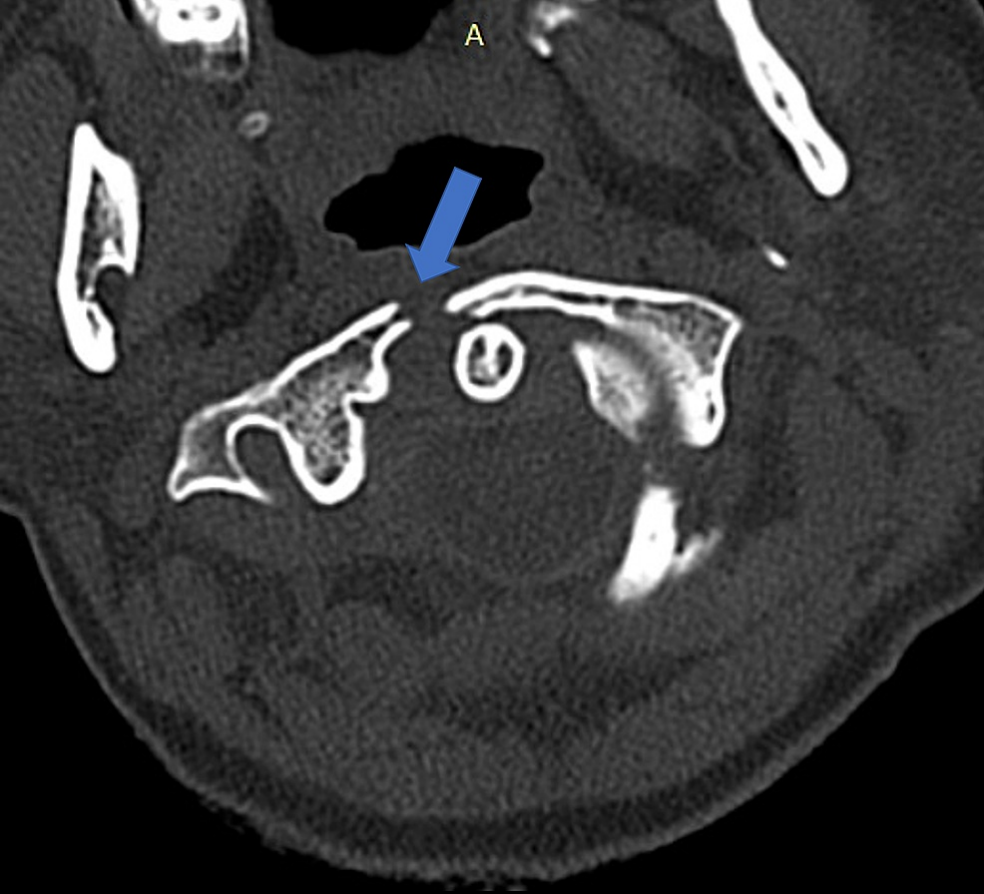
cervical CT axial view: increased angulation of the right C1 anterior arch fracture (blue arrow)

The patient also was found to have a left pneumothorax, pleural effusion and pulmonary contusions. Also, liver and spleen lacerations were detected which did not require surgery. The patient was followed-up in the ICU for a week with a cervical collar and lumbar brace. After she was stable and extubated, she did not show any motor or sensory deficits. As the patient still had moderate pleural effusion and pulmonary contusions, posterior approaches were avoided at first so that a C6-T1 anterior stabilization with mini plate-screws was performed (Figure 1D). After her pulmonary problems resolved, a second baseline CT was scheduled in which the angulation of the C1 fracture was found to have increased (Figure 2).

**Figure 3:**
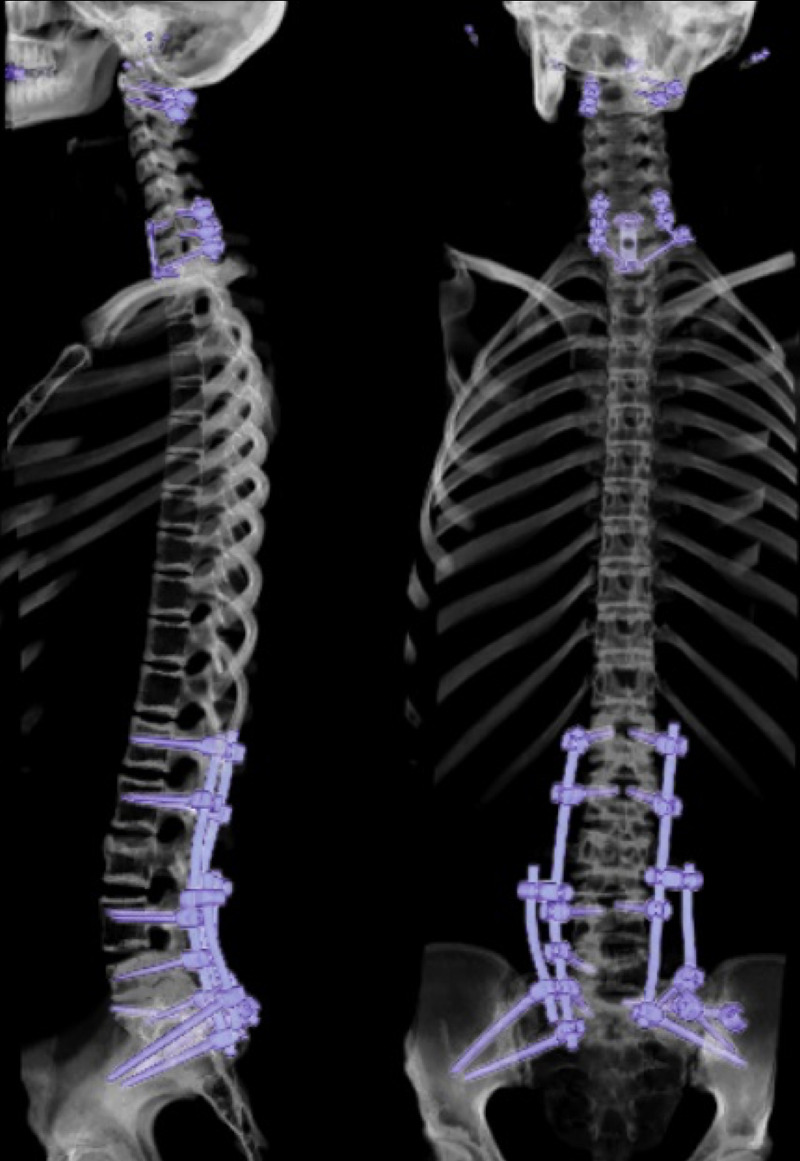
postoperative CT-3D imaging. C1 lateral mass screw combined with C2 pedicle screws, C6-T1 anterior stabilization with mini-plate screws and C6-C7 lateral mass and T1 transpedicular screws, Placement of bilateral transpedicular screws and rods was carried out at L1, L2 and L4 and on the left at L5. At S1 and S2 sacrum-alar and iliac screws and percutaneous sacral screws were also inserted

A series of spinal instrumentation surgeries were performed the during the following weeks. At first C1 lateral mass screws combined with C2 pedicle screws were inserted to stabilize her right C1 arcus fracture. Next, the anterior fixation from the first surgery was reinforced by inserting C6-C7 lateral mass and T1 transpedicular screws. In another operation, her lumbar spine was stabilized via placement of transpedicular screws and rods bilaterally at L1, L2 and L4 and on the left at L5. Insertion of S1 and S2 sacrum-alar and iliac screws and rods and the percutaneous placement of sacral screws was also carried out (Figure 3). 

The patient did not show any motor or sensory deficits after the surgeries. She was transferred to the general ward on postoperative day three and mobilized and released from hospital on postoperative day 10 without any complaints.

## Discussion

Multiple vertebral fractures occurring in a single case that need operative intervention are quite rare. Since some minor fractures like transverse process and anterior teardrop do not cause neurological deficits, it is nearly impossible to estimate their exact incidence. Approximately 33-60% are unrecognized and are only detected incidentally on radiographic images in osteoporotic patients or those on glucocorticoid therapy, which also leads to a decreased bone density [6-8]. Our patient was admitted to our clinic with multiple junctional fractures that all required surgery, which has not been reported in the literature to date. The patient's critical general status on admission and her pulmonary injuries were the primary factors that formed the algorithms for our further surgical plans. As the patient's general neurological condition improved but since she had pulmonary effusion, a posterior approach was not the primary option. Neagely et al. reported in their study that these patients had markedly decreased oxygen saturation when put prone [9]. To avoid risking any further respiratory complications, an anterior approach was done first. Her C7 fracture was classified as A2 according to the AO Spine subaxial classification system [10]. Accepted as unstable, a temporary anterior fixation with mini-plate screws was performed until posterior interventions could be done. After the effusion in her lungs resolved, she was able to be put in a prone position and posterior approaches could be initiated. On admission, the patient’s C1 fracture line went through the right anterior arch, which is defined as a Jefferson type 2 fracture (Figure 1), which is considered stable and did not need surgical intervention. Despite wearing a cervical hard collar, her neck movements could not be restricted due to her agitated psychological status. On the follow-up CT an avulsion of the right anterior arch was detected and was defined as a Jefferson type 5 fracture and assumed to be unstable; they require the surgery which the patient underwent (Figure 2) [11]. The L3 fracture was an incomplete burst fracture (A3) and the L5 fracture a complete burst fracture (A4), which according to the AO Spine classification system both needed surgical intervention [10]. The patient’s sacral fracture was considered a Denis zone 2 fracture with a shear component, which is accepted as highly unstable and required surgical fixation [12]. The patient was released from the hospital without any neurological deficits. She was able to go back to school and live her normal sociocultural life. She also got psychological support due to her suicide attempt. A further surgery is planned to remove the sacrum screws in the following years after fusion is confirmed on radiological images to avoid any complications during delivery if she decides to get pregnant.

## Conclusions

It is important in a case like this to emphasize if and when surgical intervention is needed. A multidisciplinary assessment of trauma surgeons, neurosurgeons and anesthesiologists is vital for forming a further treatment plan. The patient’s general condition and neurological status were the primary factors determining further steps. In a comatose patient who is not able to be mobilized, which decreases predicted life expectancy, there is no real conclusion if major instrumentation or immobilization with collars is indicated. After our patient’s general condition improved, we considered surgery. Her pulmonary injuries restricted posterior interventions from prone and after careful assessment and close communication between the neurosurgeon and anesthesiologist an anterior approach was considered at the beginning. After her pulmonary status improved the patient was capable for posterior interventions.

Our case might cause some controversy if all these instrumentation surgeries were necessary. But several guidelines for determining the stability of the fracture and need of surgical invention like the AO Spine classification system for subaxial fractures, Jefferson classification for C1 fractures and Denis classification for sacrum fractures must be taken notice of, and according to them all interventions were necessary.
